# Dietary Lycopene Supplementation Could Alleviate Aflatoxin B_1_ Induced Intestinal Damage through Improving Immune Function and Anti-Oxidant Capacity in Broilers

**DOI:** 10.3390/ani11113165

**Published:** 2021-11-05

**Authors:** Md Touhiduzzaman Sarker, Xiaoli Wan, Haiming Yang, Zhiyue Wang

**Affiliations:** College of Animal Science and Technology, Yangzhou University, No. 48 Wenhui East Road, Yangzhou 225009, China; sarker_06@yahoo.com (M.T.S.); wanxl1021@126.com (X.W.); hmyang@yzu.edu.cn (H.Y.)

**Keywords:** aflatoxinB1, lycopene, immune function, antioxidant, intestine, broiler

## Abstract

**Simple Summary:**

Aflatoxin B_1_ (AFB_1_) is a common and devastating food-borne fungal toxic in the broiler industry. AFB_1_ has adverse effects on the world poultry industry as it impairs the health, performance, intestinal integrity, and immunity of broilers. There has been a great economic concern in the global poultry production, leading to billions of dollars loss every year due to AFB_1_. Antibiotic-free broiler production relies on feed supplementation as an alternative to antibiotic usage. We previously reported that dietary supplementation with lycopene (LYC) has shown a promising effect on performance and intestinal integrity in the broilers infected with AFB_1_. However, there are few reports on the effects of LYC on the intestinal development and antioxidant capacity in the broiler against AFB_1_. The present study was conducted to evaluate the effects of LYC supplementation on the intestinal immune function, barrier function, and antioxidant capacity of broilers fed with an AFB_1_ contaminated diet. The findings of this research highlighted that the diet supplemented with LYC has a potential effect to improve intestinal health and reduce AFB_1_ related oxidative and inflammatory status. Hence, this study suggested that addition of LYC in broiler poultry production alleviates the AFB_1_ toxicity.

**Abstract:**

The present study aims to evaluate the effects of lycopene (LYC) supplementation on the intestinal immune function, barrier function, and antioxidant capacity of broilers fed with aflatoxinB1 (AFB_1_) contaminated diet. A total of 144 one-day-old male Arbor Acres broilers were randomly divided into three dietary treatment groups; each group consisted of six replicates (eight birds in each cage). Treatments were: (1) a basal diet containing neither AFB_1_ nor LYC (Control), (2) basal diet containing 100 µg/kg AFB_1_, and (3) basal diets with 100 µg/kg AFB_1_ and 200 mg/kg LYC (AFB_1_ and LYC). The results showed that dietary LYC supplementation ameliorated the AFB_1_ induced broiler intestinal changes by decreasing the inflammatory cytokines interferon-γ (IFN-γ), interleukin 1beta (IL-1β), and increasing mRNA abundances of cludin-1 (CLDN-1) and zonula occludens-1 (ZO-1) in the jejunum mucosa. On the other hand, AFB_1_-induced increases in serum diamine oxidase (DAO) activities, D-lactate concentration, mucosal malondialdehyde (MDA), and hydrogen peroxide (H_2_O_2_) concentrations were reversed by dietary LYC supplementation (*p* < 0.05). Additionally, LYC supplementation ameliorated the redox balance through increasing the antioxidant enzyme activities and their related mRNA expression abundances compared to AFB_1_ exposed broilers. In conclusion, dietary supplementation with LYC could alleviate AFB_1_ induced broiler intestinal immune function and barrier function damage and improve antioxidants status.

## 1. Introduction

The small intestine is a physiological and mechanical barrier consisting of intestinal epithelial and tight junction proteins. It is essential for protecting the gut organs from pathogens, toxins, and other potentially hazardous substances, and forms a structure to preserve the intestinal epithelial and barrier function [1]. AFB_1_ is produced by fungi (Aspergillus flavus and Aspergillus parasiticus) and is a common contaminated poultry feed [2]. AFB_1_ can also compromise the fundamental intestinal function, and disruption of intestinal epithelial results in leaky gut, contributing to bacterial translocation [3,4,5]. When tight junction proteins claudin (CLDN), occludin (OCLN), and zonula occludens-1 (ZO-1) are affected by aflatoxinB1 (AFB_1_), more translocation of bacteria and toxins will take place, resulting in increased oxidative response and a state of inflammation. It enhances the generation of free radicals, prolongs oxidative damage, and leads to cell damage and, finally, animal death [6,7]. As oxidative stress has a significant impact on the AFB_1_ toxicity mechanism, adding antioxidants (e.g., lycopene and curcumin) to animals’ diets increased their antioxidant and immunity system, protecting them against AFB_1_ toxicity [8,9,10]. Dietary treatments, such as lycopene (LYC) supplementation and derived items high in antioxidants have been shown to reduce the harmful effects of AFB_1_ contamination on birds by reducing the detrimental consequences of intestinal damage [11,12,13].

Lycopene is among the most effective antioxidants in the carotenoid family; its activity against biological reactive oxygen species (ROS) may prevent or alleviate oxidative damage to tissues and cells in animals [14]. It has been shown that LYC supplementation could improve antioxidant capacity and regulate lipid metabolism in chickens [15,16]. In numerous epidemiological studies, LYC has been shown to have anti-inflammatory, anti-cancer, anti-cardiovascular disease, and detoxifying properties [17,18,19,20]. Recently, Sahin et al. [21] reported that LYC activated antioxidant enzymes and nuclear transcription factor systems in heat-stressed broilers. According to Rivas et al. [22], an LYC-rich diet may help to reduce oxidative stress, restrict the detrimental effects of ROS on bone cells, and prevent osteoporosis. LYC also protected lymphocytes against oxidative stress and improved immune function [23]. Compared with other carotenoids (β-carotene, lutein, and zeaxanthin), LYC is the most efficient singlet oxygen quencher [24]. However, the majority of broiler research have focused on the impacts of dietary LYC supplementation on broiler performance and metabolism [16,25,26]. It remains uncertain whether LYC has a regulatory role in the inflammatory and oxidative stress of the broiler gut. Based on prior investigations, we assume that dietary LYC supplements can ameliorate intestinal injury in broilers. The purpose of this research was to find out more about the AFB_1_ induced intestinal damage and investigate whether LYC supplementation exerts anti-inflammatory and antioxidant effects on the intestinal damages in the AFB_1_ contaminated broilers.

## 2. Materials and Methods

### 2.1. Ethical Statement

This experiment was conducted in the College of Animal Science and Technology, Yangzhou University, China, and all protocols were approved by the Yangzhou University animal care and ethics committee (Approval Number: SYXK (Su) 2016-0020).

### 2.2. Experimental Birds, Diets, and Management

A total of 144 one-day-old male Arbor Acres broilers were obtained from a commercial hatchery (Nantong, Jiangsu Province, China). The broiler chicks were indiscriminately allocated to 3 dietary treatment groups; each group comprised 6 replicates (cages) of 8 broilers in each replicate. Treatments were (1) a basal diet containing neither AFB_1_ nor LYC (Control), (2) basal diet containing 100 µg/kg AFB_1_, and (3) basal diets supplemented with 100 µg/kg AFB_1_ and 200 mg/kg LYC (AFB_1_ + LYC). In an environmentally controlled facility house, all birds were reared under a cage breeding system. The breeding house temperature was 32–34 °C for the first 3 days, and then subsequently decreased 2–3 °C every week until the final temperature was achieved at 21 ± 1 °C during the 42-d experimental trial. During the trial, all broilers had unrestricted access to mash feed and fresh water, and the lighting cycle was 23 h of light and 1 h of darkness. Corn-soybean-based diets were developed in accordance with NRC [27] to fulfill the nutritional needs for broilers throughout the 1–21 day (starter) and 22–42 day (grower) experimental periods (Table 1).

Aflatoxin B_1_ (Purity ≥ 98%, HPLC) and Lycopene (Purity ≥ 80%, HPLC) were purchased from Shanghai Yuanye Biotechnology Co. Ltd. (Shanghai, China). The effective dose of AFB_1_ in the broiler diets and LYC supplementation was optimized according to previous studies [21,28,29]. 

### 2.3. Collection of Samples and Measurement 

The blood samples were collected in tubes by puncture of the brachial vein. The serum was extracted from the blood samples and then stored at −70 °C for subsequent analysis after centrifugation (cence DL-5M) at 3500 rpm for 10 min at 4 °C. After obtaining blood samples, the broilers were euthanized by cervical dislocation, and the small intestine segments (jejunum) were promptly removed. Afterward, the intestine segments were opened longitudinally and flushed with ice-cold sodium chloride saline. The jejunal mucosa was collected into sterile plastic tubes by scraping a sterile glass microscope slide. The mucosal samples were promptly frozen in liquid nitrogen and kept at −70 °C for further examination.

### 2.4. Preparation of Intestinal Mucosal Homogenate

Approximately 0.2 g of intestinal tissue samples (jejunum mucosa) were weighed and homogenized with cold sodium chloride saline solution (4 °C) at a ratio of 1:9 (wt/vol) using an ultraturrax homogenizer (Shanghai Jing Xin). The homogenized solution was then centrifuged for 10 min at 4 °C at 2500 rpm, and the supernatant was collected and kept at −20 °C for future analysis (see below).

### 2.5. Assay of Mucosal Inflammatory, Serum D-Lactate, and Diamine Oxidase (DAO) Status

The concentrations of interleukin-1β (IL-1β), interleukin -10 (IL-10), and interferon-γ (IFN-γ), and serum D-lactate concentration, and diamine oxidase (DAO) activities were measured using a spectrophotometric method. The commercial enzyme-linked immunosorbent assays (ELISA) kits (Nanjing Jian Cheng Bioengineering Institute, Nanjing, China) were used to determine the concentrations of the jejunal mucosa according to the manufacture instruction: add 100 µL of tissue samples in sample dilution buffer per well at 96-well microplate; cover the plate with foil paper and incubate it for 30 min at 37 °C; after that, remove the liquid and wash the plate 3 times at 300 µL of washing solution; add 100 µL conjugate solution to each well and incubate for 30 min at 21 °C; repeat the wash 3 times at 300 µL of washing solution; add 100 µL of substrate solution to each well and incubate for 15 min at 21 °C in the dark to properly cover the plate with aluminum foil paper; finally, add 100 µL stop solution to each well to stop the reaction. Determine the optical density (OD) of each well immediately, using the spectrophotometer to 450 nm. 

### 2.6. Assay of Antioxidant Parameters

According to the manufacturer’s instructions, the antioxidant parameters in the jejunal mucosa were determined using commercial kits (Nanjing Jian Cheng Bioengineering Institute, Nanjing, China). Glutathione-S-transferase (GSH-ST, catalog No. A004) was assayed by 0.1 µL of homogenate tissue samples and mixed with 0.3 µL matrix buffer to constitute the reaction mixture and incubated 37°C at 10 min. The mixture was then added to a working solution and dehydrate alcohol 1 mL, respectively, and centrifuged 3500 rpm for 10 min then the absorbance was determined at 412 nm. Similarly constituted reaction mixture without the samples containing the enzyme served as a reference blank. The activity of Glutathione peroxidase (GSH-PX, catalog No. A005) was assayed by 0.2 µL of homogenate tissue samples, mixed with 0.3 µL 1 mmol/L GSH solution and working solution 0.2 µL to constitute the reaction mixture, and incubated at 37 °C for 5 min. The mixture was then added reagent working solution 1 mL and centrifuged 3500 rpm for 10 min then the absorbance was determined at 412 nm. The activity of Catalase (CAT, catalog No. A007-1-1) was assayed by splits hydrogen peroxide, resulted in the loss of absorbance, and was determined at 405 nm. The activity of glutathione reductase (GR, catalog No. A062-1-1) was assayed by 20 µL of homogenate tissue samples was mixed with a 2.4 mL working solution to constitute the reaction mixture. After that, the absorbance was determined at 340 nm for 30 s and then incubated at 37 °C for 2 min, before again measuring the absorbance at 340 nm for 2 min 30 s. The concentration of glutathione (GSH, catalog No. A064-2-1) was assayed by 100 µL of homogenate tissue samples and mixed with 100 µL and 12.5 µL reagents, respectively, to constitute the reaction mixture, incubated at 5 min in room temperature, and then the absorbance was determined at 405 nm. A similarly constituted reaction mixture, without the samples, served as a reference blank. The concentration of malondialdehyde (MDA, catalog No. A003-1) was assayed by 50 µL of homogenate tissue samples and mixed with 1500 µL and 50 µL reagents, respectively, to constitute the reaction mixture, and 500 µL 50% glacial acetic acid, then incubated at 95 °C for 40 min. After that, it was cooled with tap water and centrifuged 3500 rpm for 10 min, then the absorbance was determined at 532 nm. The concentration of hydrogen peroxide (H_2_O_2_, catalog No. A064-1-1) was assayed by 100 µL of homogenate tissue samples and mixed with 1 mL reagent to constitute the reaction mixture, then the absorbance at was determined 405 nm. The concentration of total protein (TP, catalog No. A045-2) was assayed by 500 µL of homogenate tissue samples mixed with 3 mL Coomassie brilliant blue reagent to constitute the reaction mixture, then the absorbance was determined at 595 nm using a spectrophotometer (Thermo scientific multiskan FC, Shanghai, China). All findings from the intestinal mucosa were standardized against the total protein concentration.

### 2.7. Total RNA Extraction and mRNA Quantification

According to the manufacturer’s instruction, the total RNA of the jejunum mucosa sample was isolated using the TRIzol reagent kit (Tiangen Biotechnology, Beijing, China). The purity and concentration of extracted RNA were evaluated using the Nanodrop ND-1000 UV-spectrophotometer and electrophoresis on 1 percent agarose gel (Nano Drop Technologies, Wilmington, DE, USA). The RNA samples within the 260/280 OD ratios 1.8–2.0 were chosen for further reaction. Samples of RNA were diluted with RNase-free water to the concentration of 0.5 µg/µL. After dilution, 1 µg of that total RNA was reverse-transcribed into complementary DNA (cDNA), a 1st standard cDNA synthesis super Mix for qPCR reagent kits with a gDNA digesta plus (Yeasen Biotechnology Co. Ltd. Shanghai, China) was following the manufacturer instruction. Real-time PCR was performed using the Hifair supermix (Yeasen Biotechnology Co. Ltd. Shanghai, China) on the Quant Studio 5 Real-Time PCR system (Thermo Scientific, Wilmington, NC, USA). To normalize the expression of the other target genes, the β-actin gene was chosen as the housekeeping gene. The primers were synthesized by Yeasen Biotech (Yeasen Biotechnology Co. Ltd. Shanghai, China), and the primer sequences are presented in Table 2. The reaction mixture comprised of 2 µL of cDNA template, 0.4 µL each of the forward and reverse primers, 10 µL of SYBR master mix (Yeasen Biotechnology Co. Ltd. Shanghai, China), 0.4 µL of ROX reference dye, and 6.8 µL of double-distilled water. The PCR reaction program cycling parameters consisted of a pre-run at 95 °C for 30 s, 40 cycles at 95 °C for 5 s, followed by a 60 for 30 s with a final melting temperature at 95°C for 10 s, followed by an increase in temperature from 65 to 95 °C with a temperature change rate of 0.5 °C/s. The relative mRNA expression levels of target genes (fold changes) were analyzed by the 2^−ΔΔCt^ technique [30] after normalization against the reference housekeeping gene β-actin. The mRNA abundance of each target gene of broilers in the control group was assigned a value of 1.

### 2.8. Statistical Analysis

Statistical analysis of all data was performed by one-way ANOVA using SPSS software (version 21, SPSS, Inc., Armonk, New York, USA). Differences among the group were examined using Duncan’s multiple range test. Statistical significance was considered at *p* < 0.05, and 0.05 < *p* < 0.10 defined as a tendency towards significance. All results are expressed as the mean and standard error of the means (SEM). 

## 3. Results

### 3.1. Inflammatory Cytokines Concentration of the Intestine Mucosa

Compared with the control group, AFB_1_ contaminated broiler had a significantly higher (*p* < 0.05) concentration of IFN-γ at 21 and 42 d, and lower IL-10 at 21 d in the jejunum mucosa (Table 3). The increased mucosal IFN-γ concentration of AFB_1_ contaminated group broilers was reduced, and decreased IL-10 concentration was increased by dietary LYC supplementation.

### 3.2. Inflammatory Related Gene Expression of the Intestine Mucosa

**Figure 1:**
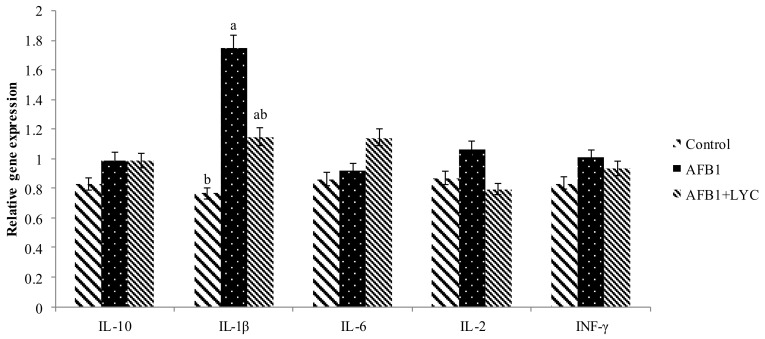
Effects of LYC on inflammatory gene expression level in the jejunum of broiler intestine fed AFB_1_ contaminated diets at 21 and 42 d. The column and its bar represented the means and standard error of the means (*n* = 6), respectively. ^a, b^ Means with different superscript letters are significantly different (*p* < 0.05). IL-10, interleukin-10; IL-1β, interleukin-1beta; IL-6, interleukin-6; IL-2, interleukin-2; INF- γ, interferon-γ; AFB_1_, AflatoxinB _1_; LYC, Lycopene; and AFB_1_ + LYC, (100 µg/kg AFB_1_ + 200 mg/kg LYC).

Compared with the control group, AFB_1_ contaminated broilers diet tended to increase (*p* = 0.061) mRNA levels of IL-1β. In addition, dietary LYC supplementation had no effects on altered mRNA levels. On the other hand, AFB_1_ contaminated diet and dietary LYC supplementation did not alter mRNA levels on IL-10, IL-6 IL-2, and INF-γ in the jejunum mucosa (Figure 1).

### 3.3. D-Lactate Concentration and DAO Activity in Serum

**Figure 2:**
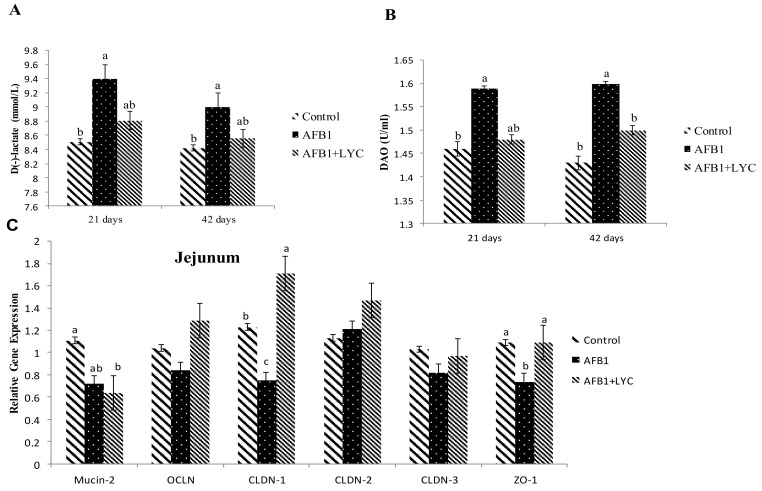
Effects of LYC on Serum D-lactate concentration (**A**) and diamine oxidase activities (**B**) in the broiler intestine fed AFB_1_ contaminated diet at 21 and 42 d. (**C**) Effects of LYC on tight junction related gene expression in the jejunum of broiler intestine fed AFB_1_ contaminated diets. The column and its bar represented the means and standard error of the means (*n* = 6), respectively. ^abc^ Means with different superscript letters are significantly different (*p* < 0.05). DAO, diamine oxidase; Mucin-2, mucins-2; OCLN, occludin; CLDN-1, claudin-1; CLDN-2, claudin-2; CLDN-3, claudin-3; ZO-1, zonula occludens-1; AFB_1_, AflatoxinB _1_; LYC, Lycopene; and AFB_1_ + LYC, (100 µg/kg AFB_1_ + 200 mg/kg LYC).

AFB_1_-contaminated broilers showed significantly greater (*p* < 0.05) serum D-lactate concentrations at 21 d (Figure 2A) and DAO activity at 42 d (Figure 2B) as compared to the control group. However, compared to the control group, the D-lactate concentration at 42 d (*p* = 0.092) and the DAO activities at 21 d tended to increase (*p* = 0.077) in the AFB_1_ broiler group. The administration of dietary LYC significantly reduced (*p* < 0.05) serum DAO activities compared to the AFB_1_ contaminated broiler group.

### 3.4. Tight Junction Related Genes Expression of the Intestine Mucosa

CLDN-1 and ZO-1 mRNA abundances in the jejunal mucosa were significantly lower (*p* < 0.05) in the AFB_1_ group compared to the control group (Figure 2C). In addition, supplementation with LYC significantly increased (*p* < 0.05) the expression of CLDN-1 and ZO-1 in the jejunal mucosa of the broiler intestine. There was no effect of the AFB_1_ toxic contamination or dietary LYC supplementation on the expression of OCLN, CLDN2, and CLDN3 in the jejunum mucosa (Figure 2C).

### 3.5. Oxidative Status of the Intestine Mucosa

The findings in Table 4 indicated that the jejunal H_2_O_2_ and MDA concentrations in the AFB_1_-induced group were substantially higher (*p* < 0.05) than those in the control group at 21 and 42 d. However, dietary LYC supplementation significantly decreased (*p* < 0.05) H_2_O_2_ and MDA concentration in the jejunum mucosa compared to the AFB_1_ group.

### 3.6. Antioxidant Enzyme Activities of the Intestine Mucosa

Broilers fed a diet contaminated with AFB_1_ had significantly decreased (*p* < 0.05) CAT at 21 d and GSH, GST, GSH-Px, GR, and CAT at 42 d in the jejunum as compared with those in the control group. On the other hand, dietary LYC supplementation significantly increased (*p* < 0.05) GSH, GST, and GR antioxidant enzyme activities as compared to the AFB_1_ group (Table 5).

### 3.7. Antioxidant Related Gene Expression of the Intestine Mucosa

**Figure 3:**
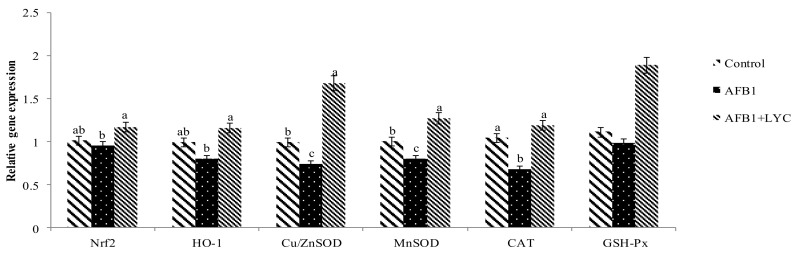
Effects of LYC on antioxidant gene expression in the jejunum of broiler intestine fed AFB_1_ contaminated diet. The column and its bar represented the means and standard error of the means (*n* = 6), respectively. ^abc^ Means with different superscript letters are significantly different (*p* < 0.05). Nrf2, nuclear factor-erythroid2-related factor2; HO-1, heme oxygenase-1; Cu/ZnSOD copper and zinc superoxide dismutase; MnSOD, Manganese superoxide dismutase; CAT, catalase; GSH-Px, glutathione peroxidase; AFB_1_, AflatoxinB _1_; LYC, Lycopene; and AFB_1_ + LYC, (100 µg/kg AFB_1_ + 200 mg/kg LYC).

Compared with the control group, the AFB_1_ toxic effect significantly decreased (*p* < 0.05) the mRNA gene expression of Cu/ZnSOD, MnSOD, and CAT in the jejunal mucosa (Figure 3). In addition, supplementation with dietary LYC significantly increased (*p* < 0.05) the expression of HO-1, Cu/ZnSOD, MnSOD, and CAT compared with the AFB_1_ group in the jejunal mucosa of broilers. The expression of jejunual Nrf2 (*p* = 0.086) trended to be increased by dietary LYC supplementation.

## 4. Discussion

The intestinal epithelium serves as a significant physical barrier to external antigens and pathogens, and disturbances are becoming more linked to infectious and non-infectious disorders [31]. A large body of research has shown that AFB_1_ contamination can affect the intestinal barrier of broilers through toxic and pathological challenges, resulting in the reduction in growth and performance [32,33,34]. In our previous study we reported that dietary supplementation with lycopene (LYC) has shown a promising effect on growth performance and intestinal integrity in the broilers infected with AFB_1_ [35]. In the current study, AFB_1_ toxic contamination increased IFN-γ and decreased IL-10 concentration in the jejunum mucosa of the broiler’s intestine, suggesting that AFB_1_-induced inflammatory response. Our findings are consistent with previous research suggesting that AFB_1_ might cause inflammation response [36,37]. The possible explanation might be that the AFB_1_ induces significant alterations in inflammatory mediator cytokines that may ultimately affect intestinal barrier function. Several types of research have revealed that the integrity of the intestine barrier can be altered by different inflammatory cytokines, such as IL-1β, INF-γ, and IL-10 [38,39]. The pro-inflammatory cytokine INF-γ concentration could increase a cellular permeability by decreasing the tight junction protein ZO-1 and CLDN1 levels in the AFB_1_ infected broilers. In addition, IL-10 antagonizes the effects of pro-inflammatory cytokines such as INF-γ and IL-1β on the tight junction protein, which leads to decreased intestinal permeability [40]. Therefore, we determined the mRNA levels of inflammatory cytokines of the intestinal mucosa, which can reflect the broiler’s inflammatory status. We demonstrated in our present study that AFB_1_ contamination increased the mRNA expression levels of IL-1β that influence the inflammatory status of the jejunum mucosa in the broilers’ intestine.

In the present study, our results showed that dietary LYC supplementation significantly decreased INF-γ concentration while increased IL-10 accumulation in the intestinal mucosa of AFB_1_ contaminated broilers. Similarly, Hashem et al. [41] revealed that LYC could prevent inflammation in rats by stimulating the production of anti-inflammatory cytokines such as IL-10 through its strong antioxidant property during colitis. Thus, results indicated that LYC supplementation could attenuate AFB_1_-induced inflammation via enhancing anti-inflammatory properties, suppressing nuclear factor-kappa B (NF-κB) [42] and activating the Nrf2/HO-1 pathway in broilers. A study by Luo and Wu [43] found that LYC treatment led to significant increases in serum IL-10 levels in rats with gastric cancer. It is generally known that IL-10, a potent anti-inflammatory cytokine, suppresses the production of pro-inflammatory cytokines, and mitigates immunological responses [44]. In addition, LYC decreased adhesion molecules, pro-inflammatory cytokines, and genes involved in inflammation [45]. These modifications could be attributed to the fact that cytokines are essential components of the host defense system and play a crucial role in defending against bacterial infections [46]. 

The intestinal epithelial barrier performs as an intestinal permeability; its function is to absorb the digestive nutrient, electrolytes, and water, and serve as a natural defense against the toxin, antigens, and enteric pathogens that are crucial for the bird’s health and growth [47]. AFB_1_ contamination may increase the permeability of the intestinal epithelial layer, resulting in excessive and uncontrolled passage of pathogenic and foreign material entering the broilers, and leading to inflammatory and oxidative responses. Gao et al. [48] found decreased tight junction protein expression (CLDN-3, occluding, and ZO-1) and disrupted intestinal permeability structure in differentiated Caco-2 cells exposed to AFB_1_. In the current study, we observed that intestinal permeable barrier function was impaired by AFB_1_ contamination, as evidenced by the increased circulating D-lactate concentration, and changed tight junction mRNA abundances of CLDN1 and ZO-1 in the intestinal mucosa. Generally, serum D-lactate concentration levels are quite low in animals. Thus, D-lactate accumulation in the systematic circulation can increase intestinal permeability induced by tight junction disorder due to AFB_1_ toxic contamination. AFB_1_ enhanced intestinal permeability in broilers by increasing the expression of CLDN1 and numerous amino acid transporters [5]. The disruptions of tight junctions increased the paracellular permeability of intestinal mucosa and were considered high pathological status [49]. On the contrary, diamine oxidase (DAO) is an intracellular enzyme produced by the intestine epithelium and present in the intestinal mucosa [5]. While disruption of the intestinal structural barrier changed tight junction genes (CLDN1 and ZO-1), expression and intestinal cells undergo necrosis condition, resulting in increased circulating DAO activity [50]. DAO activity is also an index to evaluate intestinal permeability and mucosal injury. Importantly, our result showed that dietary LYC supplementation significantly decreased serum D-lactate concentration and DAO activity and upregulated the tight junction-related mRNA abundance of CLDN1 and ZO-1 in the intestine mucosal compared to that of the AFB_1_ damaged broilers. CLDN1 and ZO-1 are the most critical components in the tight junctions’ structural and functional organization and help protect against pathogenic development [51]. Our study also showed that LYC modulated the expression of CLDN1 and ZO-1 along with its anti-toxic effects.

Aflatoxin B_1_ increased ROS generation, causing them to attack cell membrane lipids and change the fluidity and permeability of the cell membrane, resulting in oxidative damage [52]. Oxidative stress is a critical element of the disruption of mucosal barrier function [53]. It has been observed that dietary AFB_1_ contamination may reduce antioxidant enzymes activities and non-enzymatic antioxidant levels in broilers [37,54,55]. Our current experimental results showed that a diet contaminated with AFB_1_ significantly enhanced the concentration of MDA and H_2_O_2_, and decreased the antioxidant enzyme activities of GSH-Px, GST, and GR, and concentration of GSH in the small intestinal mucosa of the broilers. The results of the present investigation are consistent with previous studies, which show that AFB_1_ in broilers might generate oxidative stress [8,55,56,57]. MDA concentration is determined as an index of lipid peroxidation and is one of the main symptoms of the animal’s toxicity and carcinogenesis [58,59]. H_2_O_2_ is the main component of the ROS that is responsible for redox balance. The GST, CAT, and GSH-Px are important enzymes and GSH is the non-enzymatic components of the endogenous antioxidant defense system, which protect the organs and cells against free molecule production and lipid peroxidation [60]. In addition, the presence of CAT and GSH-Px antioxidant defense component and adequate amount of GSH reduced the oxidative stress by catalyzes H_2_O_2_ to water and O_2_ [61,62]. 

However, the results indicated that dietary LYC supplementation could ameliorate intestinal MDA and H_2_O_2_ accumulation by increasing antioxidants enzyme activities, thereby reducing oxidative stress exposed due to AFB_1_ toxic contamination in the broilers. Our results are consistent with Sahin et al. [21] they found that dietary LYC increased activities of GSH-PX, CAT, and GSH concentration and decreased MDA concentration in heat stressed broilers. According to a prior study, LYC supplementation had an antioxidant effect against T-2 toxin and zearalenone-induced toxicities [14]. Another research reported the protective and therapeutic effect of LYC against oxidative stress and epididymal epithelial cell damage caused by AFB_1_ [63]. This can be explained by the fact that LYC can prevent oxidative stress and limit free radical production to protect animals by increasing the antioxidant defense system [64].

Moreover, several studies have revealed that dysregulation of the Nrf2 signaling pathway was associated with AFB_1_ induced oxidative damage [33]. The Nrf2-HO-1 pathway is widely established for its importance in redox equilibrium in cells and tissues. Our experimental results showed increased mRNA levels of HO-1 and Nrf2 in LYC supplementation group, indicating that LYC might exert an antioxidant effect by activating the Nrf2-HO-1 signaling pathway. Nrf2 is a significant transcription factor, regulate antioxidant-related genes (HO-1, Cu/ZnSOD, CAT, and MnSOD) expression and induce cellular defense mechanisms against oxidative stress [65,66]. Several studies have revealed that LYC had antioxidant and anti-inflammatory effects, and supplementation with LYC effectively fight against inflammatory-related disorders [67,68]. Therefore, the results indicated that LYC play an important role in preventing the AFB_1_-induced oxidative damage via enhancing activation mRNA abundances of Nrf2 signaling pathway.

## 5. Conclusions

Our results indicate that dietary LYC supplementation could alleviate intestinal damages of AFB_1_ contaminated broilers by ameliorating inflammatory responses and reducing the oxidative stress of the intestinal mucosa. Therefore, LYC can be used as a broiler feed supplement to improve immunological and antioxidant status.

## Figures and Tables

**Table 1:** Composition of feed ingredients (g/kg) and nutrient level (%) as-fed basis.

Items	1–21 d	22–42 d
Ingredients (g/kg)		
Corn	570.10	610.00
Soybean meal	310.00	280.00
Corn gluten meal	40.00	24.0
Soybean oil	30.00	40.0
Dicalcium phosphate	20.00	16.0
Limestone	10.20	13.0
L-Lysine	2.00	2.50
DL-Methionine	2.00	1.50
Premix ^1^	3.10	10.00
Sodium chloride	3.00	3.00
Calculated nutrient levels (%)		
Metabolizable energy (MJ/kg)	12.61	12.96
Crude protein	21.36	19.44
Calcium	1.00	0.93
Available phosphorus	0.46	0.39
Lysine	1.09	1.05
Methionine	0.56	0.47
Arginine	1.27	1.16
Methionine+ cysteine	0.91	0.80

^1^ The premix provided per kilogram of diet: vitamin A (retinyl acetate), 12,000 IU; vitamin D3 (cholecalciferol), 2500 IU; vitamin E (DL-α-tocopheryl acetate), 20 IU; menadione, 1.3 mg; thiamin, 2.2 mg; riboflavin, 8.0 mg; nicotinamide, 40 mg; choline chloride, 400 mg; calcium pantothenate, 10 mg; pyridoxine HCl, 4 mg; biotin, 0.04 mg; folic acid, 1.0 mg; vitamin B12 (cobalamin), 0.013 mg; Fe (from ferrous sulfate), 80 mg; Cu (from copper sulphate), 8.0 mg; Mn (from manganese sulphate), 110 mg; Zn(from zinc sulfate), 60 mg; I (from calcium iodate), 1.1 mg; and Se (from sodium selenite), 0.3 mg.

**Table 2:** Primer sequences for quantitative real time PCR analysis.

Gene Name ^1^	Accession ID	Primer Sequence (5′-3′)	Length bp
IL-10	NM_001004414	Forward (F): ACTATTTTCAATCCAGGGACGA	242
		Reverse (R): GCAGGTGAAGAAGCGGTGA	
IL-1β	NM_204524	F: CCGAGGAGCAGGGACTTT	133
		R: AGGACTGTGAGCGGGTGTAG	
IL-6	NM_204628	F: AATCCCTCCTCGCCAATCT	102
		R: TCACGGTCTTCTCCATAAACG	
IL-2	NM_204153	F: TGATCTTTGGCTGTATTTCGG	169
		R: TCCTGGGTCTCAGTTGGTGT	
INF-γ	NM_205149	F: AAGAACTGGACAGAGAGAAATGAGA	154
		R: CGCCATCAGGAAGGTTGTT	
Mucin-2	NM_001318434	F: AAATGTATCTGTCGCCCCTCA	121
		R: TGTCGCCATCCTTTATTGTTG	
OCLN	NM_205128	F: TCATCGCCTCCATCGTCTAC	189
		R: CGATGAGGAACCCACAGACA	
CLDN-1	NM_001013611	F: GGATGACCAGGTGAAGAAGATG	184
		R: TGCCCAGCCAATGAAGAG	
CLDN-2	NM_001277622	F: TCAACCTGCCTCCCGACA	167
		R: GATGAAGACCACCCCACCC	
CLDN-3	NM_204202	F: TTCATCGGCAACAACATCG	242
		R: GCCTTGGTGGTCTCGTCCT	
ZO-1	XM_015278975	F: CGCTAATAGAAAGGTCCAAAGG	238
		R: CTGGAATGGTCTGAAGGCTCT	
Nrf2	NM_205117	F: CACCAAAGAAAGACCCTCCTG	201
		R: CACTGAACTGCTCCTTCGACAT	
HO-1	NM_205344	F: ACGAGCAGGCGGAGAACA	170
		R: CATCGGAAAATAAACAGGAGCA	
GSH-Px	NM_001277854	F: GTTCCAGAAGTGCCAGGTGA	207
		R: CTGTAGCGGCGGAAAGGT	
Cu/ZnSOD	NM_205064	F: AAGGGAGGAGTGGCAGAAGT	210
		R: TTTCAGGTACAACGGTTAGCACT	
MnSOD	NM_204211	F: CACTCTTCCTGACCTGCCTTAC	169
		R: CACCTGAGCTGTAACATCACCTT	
CAT	NM_001031215	F: TCTTGAGTCTTCGCCCTGAG	166
		R: TGATCGGTCTTAACGTGGAAC	
β-actin	NM_205518	F: TGATATTGCTGCGCTCGTTG	183
		R: ATACCTCTTTTGCTCTGGGCTT	

^1^ IL-10, interleukin-10; IL-1β, interleukin 1beta; IL-6, interlukin-6; IL-2, interlukin-2; IFN-γ, interferon-γ; Mucin-2, mucins-2; OCLN, occludin; CLDN-1, claudin-1; CLDN-2, claudin-2; CLDN-3, claudin-3; ZO-1, zonula occludens-1; Nrf2, nuclear factor-erythroid2-related factor2; HO-1, Heme oxygenase-1; Cu/ZnSOD copper and zinc superoxide dismutase; MnSOD, manganese superoxide dismutase; CAT, catalase; GSH-Px, glutathione peroxidase; and β-actin, beta-actin.

**Table 3:** Effects of LYC on mucosal inflammatory status in the broiler jejunum fed AFB_1_ contaminated diet at 21 and 42 d.

Dietary Treatments						
Parameters	Days	Control	AFB_1_	AFB_1_ + LYC	SEM	*p*-Value
IFN-γ (ng/g protein)	21	24.08 ^b^	27.10 ^a^	23.34 ^b^	0.586	0.011
	42	24.46 ^b^	28.42 ^a^	27.16 ^a,b^	0.669	0.036
IL-1β (ng/g protein)	21	25.73	27.11	24.98	0.447	0.142
	42	26.32	27.64	27.49	0.709	0.731
IL-10 (ng/g protein)	21	10.46 ^a^	9.28 ^b^	10.38 ^a^	0.216	0.028
	42	8.36 ^b^	8.00 ^b^	9.31 ^a^	0.191	0.006

Means with different superscripts (a, b) in the same row were significantly different at *p* < 0.05, and 0.05 < *p* < 0.10 was considered to be a tendency towards significant. SEM, pooled standard error of the means (*n* = 6), IFN-γ, interferon-γ; IL-1β, interleukin 1beta; IL-10, interleukin-10; AFB_1_, AflatoxinB_1_; LYC, Lycopene; and AFB_1_ + LYC, (100 µg/kg AFB_1_ + 200 mg/kg LYC).

**Table 4:** Effects of LYC on oxidative status in the broiler jejunum fed AFB_1_ contaminated diet at 21 and 42 d.

Dietary Treatments						
Parameters	Days	Control	AFB_1_	AFB_1_ + LYC	SEM	*p*-Value
H_2_O_2_ (µmol/gprot)	21	6.70 ^b^	8.33 ^a^	7.17 ^ab^	0.271	0.030
	42	3.03 ^b^	4.80 ^a^	3.62 ^b^	0.216	<0.001
MDA (nmol/mgprot)	21	1.41 ^b^	2.33 ^a^	1.54 ^b^	0.108	<0.001
	42	1.82 ^b^	2.35 ^a^	2.00 ^b^	0.081	0.015

Means with different superscripts (a, b) in the same row were significantly different at *p* < 0.05, and 0.05 < *p* <0.10 was considered to be a tendency towards significant. SEM, pooled standard error of the means (*n* = 6), H_2_O_2_, hydrogen peroxide; MDA, malondialdehyde; AFB_1_, AflatoxinB _1_; LYC, Lycopene; and AFB_1_ + LYC, (100 µg/kg AFB_1_ + 200 mg/kg LYC).

**Table 5:** Effects of LYC on antioxidant index in the jejunum of broilers fed with AFB_1_ contaminated diets at 21 and 42 days.

Dietary Treatments						
Parameters	Days	Control	AFB_1_	AFB_1_ + LYC	SEM	*p*-Value
GSH(µmol/gprot)	21	187.51	160.33	174.83	6.503	0.348
	42	95.92 ^a^	81.38 ^b^	94.57 ^a^	2.106	0.004
GST(U/mgprot)	21	56.36	43.92	51.01	3.412	0.244
	42	40.55 ^a^	30.66 ^b^	39.33 ^a^	1.477	0.002
GSH-Px(U/gprot)	21	5.78	4.45	5.64	0.400	0.349
	42	2.10 ^a^	1.40 ^b^	1.90 ^a,b^	0.126	0.046
GR(U/gprot)	21	8.29	6.95	8.18	0.313	0.151
	42	7.68 ^a^	5.70 ^b^	6.92 ^a^	0.277	0.005
CAT(U/mgprot)	21	6.42 ^a^	4.78 ^b^	5.67 ^a,b^	0.274	0.039
	42	5.13 ^a^	4.23 ^b^	4.60 ^a,b^	0.150	0.038

Means with different superscripts (a, b) in the same row were significantly different at *p* < 0.05, and 0.05 < *p* < 0.10 was considered to be a tendency towards significant. SEM, pooled standard error of the means (*n* = 6), GSH, glutathione; GST, glutathione-S-transferase; GSH-Px, glutathione peroxidase; GR, glutathione reductase; CAT, catalase: AFB_1_, AflatoxinB_1_; LYC, Lycopene; AFB_1_, (100 µg/kg AFB_1_); and AFB_1_ + LYC, (100 µg/kg AFB_1_ + 200 mg/kg LYC).

## Data Availability

The data presented in this research study are available on request from the corresponding author.
